# Role of retinal pigment epithelium‐derived exosomes and autophagy in new blood vessel formation

**DOI:** 10.1111/jcmm.13730

**Published:** 2018-08-21

**Authors:** Sandra Atienzar‐Aroca, Gemma Serrano‐Heras, Aida Freire Valls, Carmen Ruiz de Almodovar, Maria Muriach, Jorge M. Barcia, Jose M. Garcia‐Verdugo, Francisco J. Romero, Javier Sancho‐Pelluz

**Affiliations:** ^1^ School of Medicine Catholic University of Valencia Valencia Spain; ^2^ Experimental Research Unit General University Hospital of Albacete Albacete Spain; ^3^ Heidelberg Biochemie‐Zentrum (BZH) University of Heidelberg Heidelberg Germany; ^4^ Unidad predepartamental de Medicina Universitat Jaume I Castellón de la Plana Spain; ^5^ Department of Comparative Neurobiology Universidad de Valencia Valencia Spain; ^6^ Faculty of Health Sciences Universidad Europea de Valencia Valencia Spain

**Keywords:** angiogenesis, autophagy, exosomes, oxidative stress, retina, retinal pigment epithelium, siRNA, VEGFR2

## Abstract

Autophagy and exosome secretion play important roles in a variety of physiological and disease states, including the development of age‐related macular degeneration. Previous studies have demonstrated that these cellular mechanisms share common pathways of activation. Low oxidative damage in ARPE‐19 cells, alters both autophagy and exosome biogenesis. Moreover, oxidative stress modifies the protein and genetic cargo of exosomes, possibly affecting the fate of surrounding cells. In order to understand the connection between these two mechanisms and their impact on angiogenesis, stressed ARPE‐19 cells were treated with a siRNA‐targeting Atg7, a key protein for the formation of autophagosomes. Subsequently, we observed the formation of multivesicular bodies and the release of exosomes. Released exosomes contained VEGFR2 as part of their cargo. This receptor for VEGF—which is critical for the development of new blood vessels—was higher in exosome populations released from stressed ARPE‐19. While stressed exosomes enhanced tube formation, exosomes became ineffective after silencing VEGFR2 in ARPE‐19 cells and were, consequently, unable to influence angiogenesis. Moreover, vessel sprouting in the presence of stressed exosomes seems to follow a VEGF‐independent pathway. We propose that abnormal vessel growth correlates with VEGFR2‐expressing exosomes release from stressed ARPE‐19 cells, and is directly linked to autophagy.

## 1 INTRODUCTION

The retinal pigment epithelium (RPE) is a monolayer of cells highly vulnerable to oxidative stress (OS), particularly to reactive oxygen species (ROS).^1^ It has been observed that excessive ROS formation in RPE cells can deregulate other physiological mechanisms, such as autophagy.^2^, ^3^ Autophagy is a degradative process that maintains cellular homeostasis by selectively eliminating damaged organelles, proteins and cellular debris.^4^, ^5^ Macroautophagy (from now on referred just as autophagy) eliminates damaged material creating a double‐membrane vesicle known as the autophagosome, which is delivered to the lysosome for degradation.^6^ A number of proteins are involved in the activation of autophagy, including Beclin‐1, autophagy‐related proteins (Atg) 5‐12 and microtubule‐associated protein 1A/1B‐light chain 3 (LC3), which passes from form I to form II in the autophagosome membrane.^7^ Autophagy takes place naturally in cells, but can be enhanced in response to cellular stress, such as damaged DNA and excessive ROS.^8^ Consistently, overproduction of ROS due to ethanol (EtOH)‐induced damage, generates more autophagosomes.^3^ Moreover, expression of cell death markers, such as Bax, seems to be enhanced in a concentration‐dependent manner.^2^

Communication between RPE cells, endothelial cells (ECs) of the choriocapillaris and photoreceptor cells is crucial for the proper maintenance of visual homeostasis and retinal functions. Exosomes, extracellular vesicles (EVs) which range in diameter from 30 to 100 nm,^9^ are essential in cell‐to‐cell communication as their cargo contain genetic material and a number of proteins, which may be delivered into neighbouring cells.^10^ Invaginations in the late endosome membrane fills it with intraluminal vesicles (ILV), generating a multivesicular body (MVB),^11^ which might fuse with autophagosomes or lysosomes, resulting in the degradation of the vesicles.^12^ However, when an MVB fuses with the cell membrane, it releases the ILVs, now exosomes, to the extracellular medium.^13^ Exosomes are found in most corporal fluids, including blood, saliva, breast milk and aqueous humour.^14^, ^15^, ^16^, ^17^ Under stress, RPE cells release an elevated number of exosomes, which contain a different cargo from that observed in exomes derived from healthy RPE.^17^, ^18^, ^19^

Moreover, increased production of ROS in the RPE generates overproduction of vascular endothelial growth factor (VEGF) and subsequent accelerated angiogenesis in the choroid.^20^, ^21^ We observed that stressed RPE cells release a higher fraction of exosomes with VEGF receptor 2 (VEGFR2) in their membrane. When EC cultures were treated with such exosomes, the formation of new blood vessels was accelerated.^19^

Correlations between autophagy and exosome release have been already suggested.^22^, ^23^, ^24^ Interestingly, it was proposed that the interaction between autophagic vacuoles and MVBs contributes to the removal of superfluous organelles and proteins.^25^ In fact, enhanced ROS increases the number of MVBs, which can be released or combined with lysosomes or autophagosomes, forming the amphisome, thus degrading their content.^26^ Hence, overproduction of ROS increases autophagy activity and exosome release.

Exosomes have been observed in the extracellular medium of ARPE‐19, a human RPE cell line. When ARPE‐19 cells were challenged, they released a larger number of exosomes containing pro‐autophagy factors such as Atg5‐Atg12 and Beclin‐1.^18^ Furthermore, in a model of age‐related macular degeneration (AMD), it has been proposed that exosomes released by stressed RPE cells are able to increase autophagy in other cells, and that this can contribute to drusen formation, an early symptom of AMD.^22^ It has been recently hypothesized that exosomes and autophagy work in concert to maintain cellular homeostasis.^23^ Conversely, the release of exosomes has been shown to damage neighbouring cells.^27^

In the present study, we intend to establish a link between two phenomena: autophagy and exosome release in RPE cells under OS. We also aim to study the contribution of these two cellular mechanisms to the regulation of angiogenesis. In a physiological environment, there is equilibrium between angiogenic activators and inhibitors, resulting in very limited new blood vessel formation. Nevertheless, angiogenic stimulators can break the balance in a number of visual conditions, such as AMD and diabetic retinopathy (DR).^28^, ^29^

In summary, induction of low oxidative stress in RPE cells overactivates autophagy and enhances the number of exosomes released to the extracellular medium. Under such stress conditions, the fraction of RPE exosomes containing VEGFR2 is augmented, causing ECs to migrate and form new blood vessels. Thus, abnormal blood vessel formation might be affected by exosomes released from damaged RPE.

## 2 MATERIAL AND METHODS

### 2.1 Cell culture and treatments

Human retinal pigment epithelial (ARPE‐19) cell line was obtained from the American Type Culture Collection (ATCC). ARPE‐19 cells were cultured in Dulbecco's modified Eagle's DMEM/F12 (Invitrogen, Carlsbad, CA, USA), as previously performed.^19^ Cells were used from passages higher than 20. Depending on the technique, cells were cultured in P‐100 well plate at a starting seed density of 1 × 10^6^ cells/well. After 2 days, at 80% of confluency, cells were treated for 24 hours at different EtOH concentrations. Previous studies have shown that a concentration of 80 mmol/L was sufficient to generate OS, autophagy and a peak of exosome release from ARPE‐19 cells, and that above 600 mmol/L, OS and autophagy were considerably enhanced and cells begin to die.^2^, ^3^, ^19^

Human umbilical vein endothelial cells (HUVEC) were isolated as described previously.^30^ Briefly, umbilical veins were perfused with 1% collagenase solution and incubated at 37°C for 15 minutes. Endothelial cells were recovered in specific endothelial growth medium (ENDOPAN) (Lonza, Cultek, Barcelona, Spain). For all experiments, HUVEC growth in 2% FBS growth factor‐free Endopan medium.

### 2.2 Exosome isolation and size‐distribution

First, culture supernatant was centrifuged at 700 *g* for 30 minutes (18‐20°C). The pellet was removed and the supernatant centrifuged again at 14 000 *g* for 30 minutes. Then, the supernatant was centrifuged at 40 000 *g* for 30 minutes. Subsequently, the supernatant was centrifuged at 120 000 *g* for 90 minutes (18‐20°C). Finally, the supernatant was removed and the pellet stored at 4°C until further processing. Exosome identity was confirmed by the nanoparticle tracking system NanoSight NS300 following manufacturer protocols (Malvern Instruments, Malvern, UK).

### 2.3 Protein extraction

For protein extraction, cells were treated with trypsin and lysed with RIPA buffer and protease inhibitor cocktail (Sigma‐Aldrich, St. Louis, MO, USA) and sonicated 3 cycles. Then, the samples were centrifugated at 8000 *g* during 5 minutes. The supernatant was collected and stored at −20°C until further processing. Total protein concentration was analysed with BCA Protein Assay Kit (Thermo Fisher, Waltham, MA, USA).

### 2.4 Western blot analysis

Equal amount of protein from each sample (35 mg) was measured by SDS‐PAGE on 4‐12% gels and electroblotted onto polyvinylidene difluoride membranes (Millipore, Billerica, MA, USA). Membranes were incubated overnight at 4°C with rabbit polyclonal antibodies: Bax (1:250; Santa Cruz Biotechnology, Santa Cruz, CA, USA), LC3 (1:1000; Sigma‐Aldrich), mouse monoclonal antibody against b‐actin (1:500; Santa Cruz), GAPDH (1:1000; Santa Cruz), p62 (1:1000; Cell Signaling, Boston, USA), VEGFR2 and p‐VEGFR2 (1:500 Cell Signaling) and Apg12 (1:500 Abcam, Cambridge, MA, USA). Subsequently, membranes were incubated 2 hours at room temperature (RT) in horseradish peroxidase‐conjugated anti‐mouse and anti‐rabbit IgG (1:10000; Santa Cruz). Bands were visualized with ECL (Thermo Fisher) and detected with Image Quant LAS‐4000 mini (GE Healthcare). Protein levels were quantified by densitometry using ImageJ software (National Institutes of Health). Protein expression intensity was normalized to b‐actin or GAPDH.

### 2.5 Electron microscopy

ARPE‐19 cells were seeded at a density of 3 × 10^4^ cells/well in 8‐well Lab‐Tek chamber slides (Nalge Nunc Int., Roskilde, Denmark) and fixed in 3.5% glutaraldehyde for 1 hour at 37°C. Then, cells were postfixed in 2% OsO_4_ for 1 hour at RT and stained with 2% uranyl acetate in the dark for 2 hours at 4°C. Finally, cells were rinsed in 0.1 mol/L PBS, dehydrated in EtOH and infiltrated overnight with Araldite (Durcupan, Fluka, Heidelberg, Germany). Following polymerization, serial semithin (1.5 μm) sections were cut with an Ultracut UC‐6 microtome (Leica Microsystems, Wetzlar, Germany), mounted onto slides and stained with 1% toluidine blue. Selected sections were glued (Super Glue, Loctite, Westlake, OH, USA) to araldite blocks and detached from the glass slide by repeated freezing (in liquid nitrogen) and thawing. Ultrathin (0.06‐0.09 μm) sections were prepared on the Ultracut microtome and stained with lead citrate. Calculations were made after observing 7 cells per condition.

### 2.6 Quantitative analysis of exosomes by flow cytometry

Flow cytometry is an exceptional method to detect, quantify and characterize EVs. The small size and dim signal from most vesicles challenge the sensitivity of flow cytometry. Indeed, several laboratories have reported that 0.5 μm is the cutoff value for accurately identifying extracellular vesicles using previous generation flow cytometers.^31^ However, the use of new digital flow cytometers have allowed to detect EVs below this limit, thereby providing access to measurement of vesicles subpopulations of smaller size.^32^ In the present study, we aimed to perform a quantification and characterization of surface protein expression of exosomes released from RPE cells. To this end, we used a new generation of flow cytometer, FACS Canto II (BD, Beckton Dickinson, Franklin Lakes, NJ, USA), which incorporates 2 air‐cooled lasers at 488‐ and 633‐nm wavelengths, and the BD FACSDiva TM software. Briefly, for each analysis 50 μL of isolated exosomes were suspended in 450‐μL filtered PBS and 2 μL of the corresponding non‐fluorophore‐conjugated primary antibody (VEGFR‐2, Apg12, Beclin‐1 [Abcam], p62 [Cell Signaling], Bax and Bcl‐2 [Santa Cruz]) was added and incubated for 1 hour at room temperature (RT) on rotating wheel. Subsequently, the exosomes samples were labelled by adding 2 μL of either FITC‐conjugated or PerCP‐conjugated secondary antibody (Immunostep, Salamanca, Spain) for 30 minutes at RT under rotation in the darkness. Then, exosomes were costained with APC‐conjugated antiCD9 (Abcam), an exosomal marker, during 1 hour at RT. Finally, the colabelled exosome samples were ultracentrifugated during 90 minutes at 120 000 *g* with PBS. The supernatant (containing unbound antibodies) was discard and exosomes pellets were resuspended in filtered PBS and deposited on the Flow cytometry tubes for analysis. A mix of size‐calibrated fluorescent polystyrene beads with diameters of 220, 450 and 880 nm (Spherotech Inc., Lake Forest, IL, USA) was used to select optimal instrument settings and gate. Logarithmic amplification was used for all channels (the voltages of FSC, SSC, FITC, PerCP‐Cy5‐5 and APC were 800, 450, 550, 470 and 500 Volts, respectively) and unstained exosomes were used as negative control. The upper and the outer limit of the exosomes gate was established just above the size distribution of the 220‐nm beads in a forward (FSC‐A) and side scatter (SSC‐A) setting (log scale), whereas the lower limit was the noise threshold of the instrument. In addition, a fluorescence threshold was set at 200 Volts for APC (fluorophore covalently attached to antibody that bind exosome‐specific antigen, CD9) parameter in order to separate true events from background noise caused by PBS. All samples were run with a medium flow rate of 60 μL/min and the single positive events for CD9 and double positive events for CD9‐specific surface protein were counted after 2 minutes of acquisition.

### 2.7 ATG7 silencing

Silencing mix was prepared with 500 and 2.5 μL of RNAiMAX (Thermo Fisher) per well. Subsequently, 0.75 μL of a siRNA for Atg7 20 μmol/L was added. Meanwhile, 150 000 ARPE‐19 cells/well were plated in a 6‐well plate, and 500 μL of the silencing mix were added. After 24 hours medium was changed to a normal growth medium. After 48 and 72 hours, cells and medium were collected.

### 2.8 Tube formation assay

The day before seeding the cells, the growth factor‐reduced Matrigel (BD Biosciences) was placed on ice in the refrigerator at 4°C and cultured HUVEC were starved overnight. The following day, 10 μL of gel were applied to each inner well of a μ‐slide Angiogenesis (Ibidi, Martinsried, Germany). The whole assembly was placed into the incubator for polymerization (30‐60 min). In the meantime, the cell suspension of HUVEC was prepared (PromoCell, C‐12200, C‐12203); 10 000 cells/well with starvation medium were arranged and 5 exosomes/cell were added to corresponding wells. After 4 hours, pictures were taken in a bright‐field microscope (Zeiss Axiovert 200 mol/L with 5x/0.16 Plan‐NEOFLUAR objective). Total tube length was quantified by Image J software.

When stated, 1 μg/mL recombinant Flt1/Fc (R&D Systems, 471‐F1) was added to the cell suspension to trap VEGF. After 1 hour, five exosomes/cell were added to corresponding wells. After 4 hours, pictures were taken under the microscope.

### 2.9 VEGFR2 silencing

siRNA transfection of HUVEC was performed with Oligofectamine Reagent (Invitrogen) following manufacturer's instructions. Briefly, 110 000 HUVECs/well were cultured. After 24 hours, the cells were transfected with a final siRNA concentration of 200 nmol/L. Human VEGFR2 siRNA (5′‐3′se‐GUCCCUCAGUGAUGUAGAA, as‐UUCUACAUCACUGAGGGAC) and MISSION siRNA (universal negative control) were obtained from Sigma‐Aldrich. After 4 hours of incubation, the medium was changed for 2 mL of normal growth medium.

For silencing VEGFR2 in ARPE‐19 cells the protocol was the same, but 1 × 10^6^ cells were seeded in a P100 well plate until confluency. Forty‐eight hours after the silencing process, exosomes from the medium were isolated.

### 2.10 Sprouting assay

Fibrin gel bead sprouting assay was performed as previously described.^33^ Briefly, Cytodex^®^3 microcarrier beads (GE Healthcare) were coated with siRNA‐transfected HUVECs (at ratio of 200 000 cells/1000 beads) and embedded in fibrin gels in a 24‐well plate. HUVEC‐coated beads were cultured in 2% FBS growth factor‐free Endopan medium for 24 hours. Then, 5 exosomes/cell were added to corresponding wells and incubated for 4 hours. Wells in presence or absence of VEGF were used as positive or negative control, respectively. Images were obtained with a Zeiss LSM 510 META confocal microscope (10x) and quantification was done with ImageJ.

## 3 RESULTS

### 3.1 Formation of MVBs and amphisomes after OS damage and inhibition of autophagy

**Figure 1:**
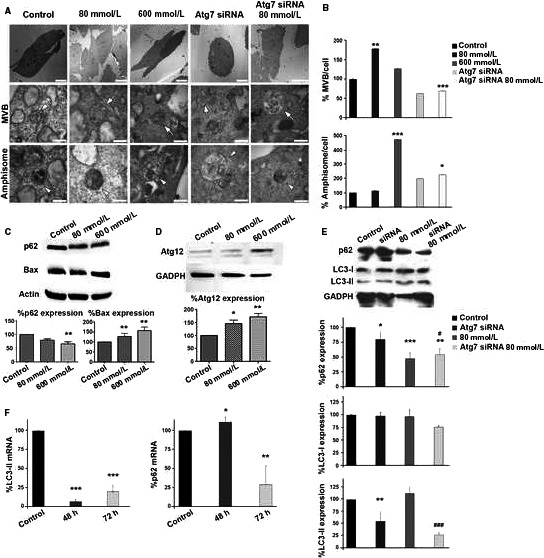
Ultrastructural changes, apoptosis and autophagy in stressed ARPE‐19 cells. A, Photographs of control and treated ARPE‐19 cells were taken under the electron microscope. Atg7 siRNA was applied to control and stressed ARPE‐19 cultures. MVBs (arrows) and amphisomes (arrowheads) were observed in every case. B, Relative quantification of MVBs and amphisomes in the aforementioned culture types. C, Relative expression levels of p62 and Bax in ARPE‐19 cells untreated, treated with low (80 mmol/L) and high (600 mmol/L) concentrations of EtOH. D, Relative expression levels of Atg12 in ARPE‐19 cells untreated and treated with low and high EtOH concentrations. siRNA‐Atg7 trial: (E) 72 hours relative protein and (F) mRNA levels of LC3‐II and p62 before and after applying siRNA‐Atg7 (for 48 and 72 hours). Scale bars: 10 μm (upper panels), 500 nm (centre and bottom panels). Values are expressed as mean ± SEM (N ≥ 3). Significance levels: (when compared to control) *P* < .05 (*), *P* < .01 (**) and *P* < .001 (***); (when compared to treated with 80 mmol/L group) *P* < .05 (#) and *P* < .001 (###)

Formation of MVBs and amphisomes were detected in control (untreated) and stressed (treated with EtOH) ARPE‐19 cells under electron microscopy (Figure 1A). Low‐stressed RPE cells (treated with 80 mmol/L EtOH) presented a greater number of MVBs than control cells, whereas the level of amphisomes was similar to that observed in untreated cells. High‐stress RPE cells (600 mmol/L) presented MVBs at control levels, but the formation of amphisomes increased more than four‐fold (Figure 1B). When ARPE‐19 cells were cultured with a siRNA targeting Atg7 (Atg7 siRNA), autophagy was repressed, less MVBs were observed in control and low‐stressed cells, and the formation of amphisomes augmented significantly in both cases (Figure 1A,B).

Expression of BAX and p62, proteins related to apoptosis and autophagy, respectively, was studied in control and stressed cells (Figure 1C). p62 was significantly reduced in high‐stressed cells, but the difference was not significant at low concentrations of treatment. Under stress, ARPE‐19 cells BAX expression increased in a concentration‐dependent manner. Expression of Atg12—a key protein for the generation of the autophagosome—was examined revealing that the level of Atg12 also increased in a concentration‐dependent manner (Figure 1D). Successful inhibition of autophagy was observed by Western blot analysis of LC3‐I and LC3‐II and p62, at 72 hours after treating with Atg7 siRNA (Figure 1E,F).

Altogether, these results suggest that low and high OS accelerates the formation of MVBs and amphisomes in ARPE‐19 cells, and that, when Atg7 is silenced, the presence of both organelles is dramatically reduced. MVBs might fuse with the membrane, releasing their content to the extracellular medium, or they can be digested by autophagosomes, forming the amphisomes. When formation of autophagosomes is blocked (by Atg7 siRNA), it seems that MVBs are disintegrated via another pathway, mostly by direct degradation by lysosomes,^23^ not being able to release their exosomes.

### 3.2 Oxidative stress and autophagy influence exosome release in RPE cells

**Figure 2:**
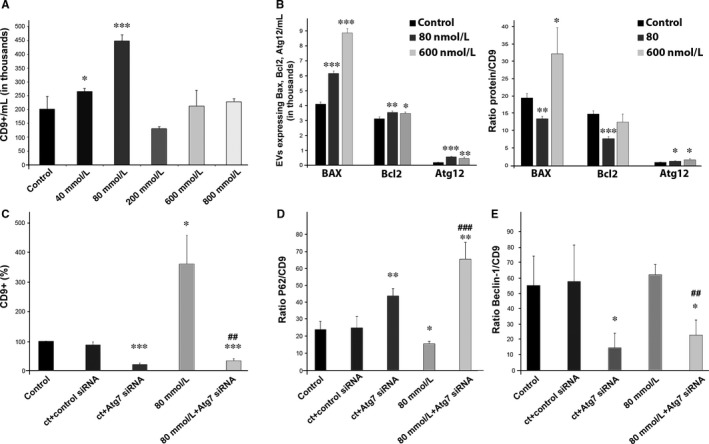
Release of EVs is increased in low‐stressed ARPE‐19 cells. A, Detection of EVs by flow cytometry. Exosomes are tracked using an antibody against CD9. B, Total number (left panel) and relative levels (right panel) of exosomes expressing Bax, Bcl‐2and Atg12 in ARPE‐19 cells untreated and treated with low and high EtOH concentration. C, Relative quantification of exosomes released from ARPE‐19 cells (control and stressed) after applying Atg7 siRNA. D, Relative levels of exosomes expressing p62, before and after applying Atg7 siRNA into control and stressed ARPE‐19 cell cultures. E, Relative levels of exosomes expressing Beclin‐1, before and after applying Atg7 siRNA into control and stressed ARPE‐19 cell cultures. Flow cytometry dot plots available in Figures S2 and S3. Values are expressed as mean ± SEM (N ≥ 3). Significance levels: (when compared to control) *P* < .05 (*), *P* < .01 (**) and *P* < .001 (***); (when compared to treated with 80 mmol/L group) *P* < .01 (##) and *P* < .001 (###)

ARPE‐19 cells released EVs which were monitored by the nanoparticle tracking system NanoSight, by observation under electron microscopy (see Figure S1), and by flow cytometry (Figures 2 and S2). OS, induced by EtOH, seemed to influence the release of EVs in ARPE‐19 cells. Apparently, at low concentrations of EtOH, the number of released exosomes increased. Moreover, a peak of exosomes was observed when cells were treated with 80 mmol/L EtOH for 24 hours (Figure 2A). At higher concentrations (200, 600 and 800 mmol/L), the release of exosomes was attenuated. These data justify the use of low concentrations of EtOH throughout the rest of the study.

Since their cargo is variable and dependent on homeostasis, exosomes released from healthy and stressed RPE cells were analysed. Exosomes containing markers for apoptosis (BAX, Bcl‐2) and autophagy (Atg12, p62 and Beclin‐1) were observed by flow cytometry and Western blot (see Figures S2, S3 and S4). Electron microscopy showed that the size of exosomes from control and stressed cells were very similar (Figure S5). The number of Bax‐positive exosomes/mL seemed to be higher in EVs released from stressed cells (Figure 2B, left panel). This might be due to the higher amount of EVs released by RPE cells when confronting OS (as aforementioned). Nevertheless, the fraction of BAX‐positive exosomes (ratio BAX/CD9) was lower when RPE cells were stressed with a low EtOH concentration, and significantly higher when cells were treated with higher concentrations (Figure 2B, right panel). The antiapoptotic protein Bcl‐2 appeared to be elevated in stressed conditions, when total EVs/mL were studied (Figure 2B, left panel). However, when low stress was applied, the Bcl‐2 fraction was significantly lower (Figure 2B, right panel). At high stress conditions, the fraction of Bcl‐2‐positive exosomes did not differ from the control set (Figure 2B). Additionally, proteins necessary for the formation of the autophagosome—Atg12, p62 and Beclin‐1—were tracked. The total amount of exosomes expressing Atg12 was increased by three‐fold in culture medium of low‐stressed ARPE‐19 cells, and by two‐fold in medium of high‐stressed cells (Figure 2B, left panel). The ratio Atg12/CD9 was also raised under low‐ and high‐stress conditions (Figure 2B, right panel).

When autophagy was inhibited by Atg7 siRNA, exosome release was also decreased, in stressed and nonstressed ARPE‐19 cells (Figure 2C). Surprisingly, the fraction of released exosomes p62‐positive increased dramatically after Atg7 interference in control and stressed RPE cells (Figure 2D). However, Beclin‐1/CD9 ratio was drastically reduced after treatment with Atg7 siRNA, whether the cells were stressed or not (Figure 2E).

Increasing evidence indicates that impaired autophagy is associated with angiogenesis, both in the development of the chicken embryo,^34^ where Atg7 plays an important role, as in the choroidal RF/6A cells, where hypoxia‐induced autophagy stimulates EC growth.^35^

**Figure 3:**
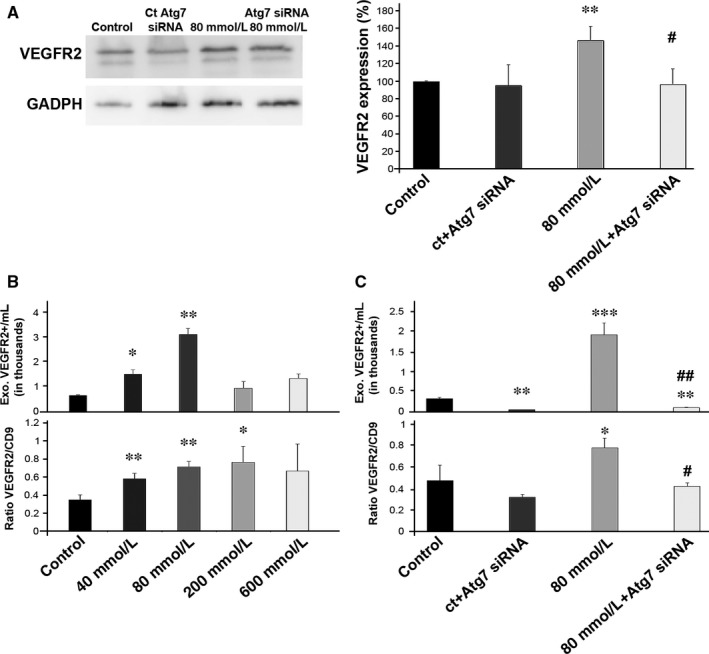
Autophagy inhibition reduced VEGFR2‐positive fraction in EVs. A, Relative quantification of VEGFR2 expression in ARPE‐19 cells (control and stressed) was studied before and after Atg7 siRNA treatment. B, Total number (upper panel) and relative levels of exosomes expressing VEGFR2 in cultures of control and stressed (40, 80, 200 and 600 mmol/L) ARPE‐19 cells. C, Total number (upper panel) and relative levels of exosomes expressing VEGFR2 in cultures of control and stressed ARPE‐19 cells, before and after applying Atg7 siRNA. Flow cytometry dot plots available in Figure S3. Values are expressed as mean ± SEM (N ≥ 3). Significance levels: (when compared to control) *P* < .05 (*), *P* < .01 (**) and *P* < .001 (***); (when compared to treated with 80 mmol/L group) *P* < .05 (#) and *P* < .01 (##)

It is already known that the stress caused by EtOH at low concentrations induces autophagy^2^ and increases the expression of VEGFR2^19^ (Figure 3A), we therefore silenced Atg7 in ARPE‐19 cells and analysed the expression of VEGFR2 both in cells and in the exosomes obtained from the medium. Autophagy inhibition by Atg7 siRNA decreased the expression of VEGFR2 in low‐stressed RPE cells (Figure 3A).

The analysis of the VEGFR2 content in exosomes showed that there is a peak of VEGFR2‐expressing exosomes when the cells were treated with 80 mmol/L EtOH (Figure 3B, upper panel). Moreover, when the ratio VEGFR2/CD9 was studied, we observed that the fraction of VEGFR2‐positive exosomes was significantly enhanced at 40, 80 and 200 mmol/L (Figure 3B, lower panel). When Atg7 siRNA was applied in control and stressed cells, there were fewer VEGFR2‐positive exosomes/mL and the percentage of exosomes carrying the protein was also considerably decreased (Figures 3C and S3C).

### 3.3 RPE‐derived exosomes and angiogenesis

**Figure 4:**
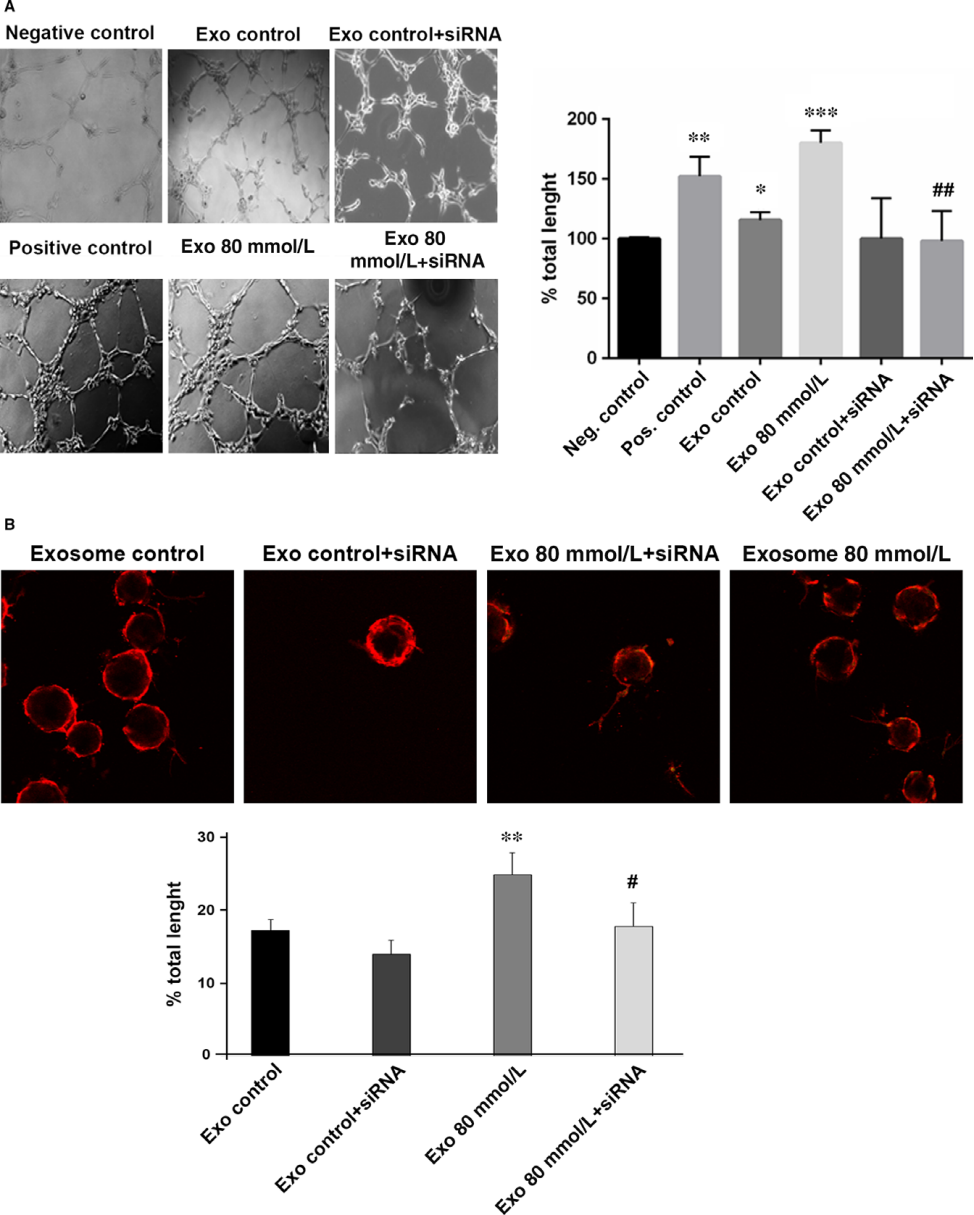
Inhibition of autophagy in RPE reduces EV‐related neovascularization in endothelial cells. A, Tube formation in HUVEC cultures after adding exosomes from ARPE‐19 cells (control and stressed) that were treated (or not) with Atg7 siRNA. Quantification of the total length of the tubes is shown in the bar chart. B, Sprouting capacity of HUVEC after 4 hours of treatment with exosomes released from ARPE‐19 cells (control and stressed), treated (or not) with Atg7 siRNA. Quantification of the total length of the sprouts is shown in the bar chart. Values are expressed as mean ± SEM (N ≥ 3). Significance levels: (when compared to control) *P* < .05 (*), *P* < .01 (**) and *P* < .001 (***); (when compared to treated with 80 mmol/L group) *P* < .05 (#) and *P* < .01 (##)

Once oxidative stress—low or high—was induced, RPE cells released a higher number of exosomes, which contained a different cargo, from the original, healthy, RPE‐derived exosomes. This new load enclosed, among other proteins, VEGF receptors that were able to fasten the generation of new blood vessels.^19^ To link OS with autophagy, and their effect on the released exosomes, we silenced Atg7 in ARPE‐19 cells, which were subsequently treated, or not, with 80 mmol/L EtOH. Released vesicles were then added to HUVECs in order to study their angiogenic capacity. Addition of exosomes released from EtOH‐treated ARPE‐19 cells, where Atg7 was knocked down, showed reduced tube formation when compared to exosomes from ARPE‐19 cells treated with control siRNA and EtOH (Figure 4A).

Sprouting angiogenesis is a fundamental mechanism in vessel growth. It is known that low‐stress conditions increased the sprouts formation in ECs.^36^ After 4 hours of treatment with exosomes in HUVEC cultures, we observed that exosomes from Atg7 siRNA‐treated cells decreased the ability to form sprouts, compared to the group treated only with low concentrations of EtOH (Figure 4B).

These findings suggest that stressed RPE derived EVs—which, as mentioned above, carry a high population of VEGFR2‐positive exosomes—are essential in the development of aberrant blood vessels.

### 3.4 Angiogenesis of HUVEC is VEGFR2‐dependent

**Figure 5:**
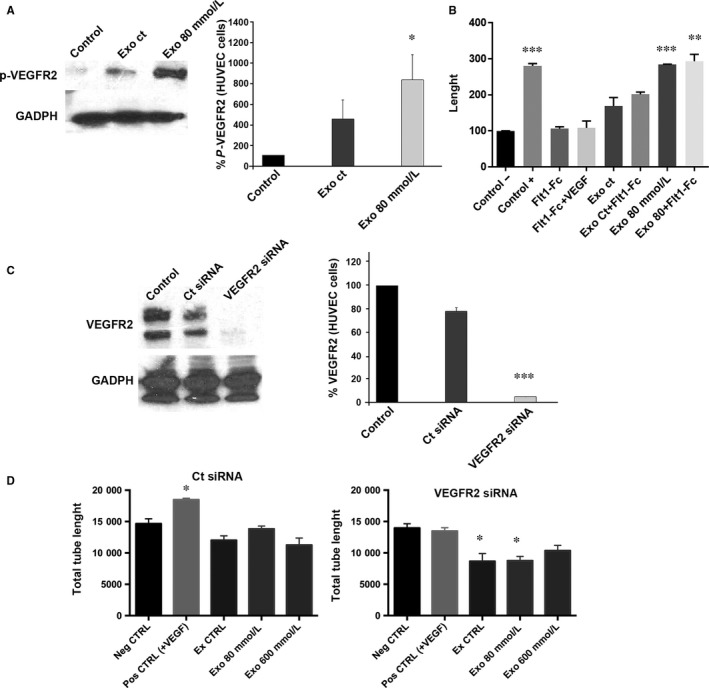
Angiogenesis of HUVEC is dependent of VEGFR2. A, Blots show expression of p‐VEGFR2 in HUVEC control, treated with exosomes from healthy RPE and treated with exosomes released from stressed RPE. The bar chart represents the relative levels of VEGFR2 expression when compared to control. B, When adding the VEGF trapper sFlt1, angiogenesis goes back to control levels, but when exosomes from stressed cells are used, sFlt1 does not arrest tube formation. C, Blots show expression of VEGFR2 in endothelial cells with or without VEGFR2 siRNA. The bar chart represents the relative levels of VEGFR2 expression when compared to control. D, When RPE‐released exosomes were added to endothelial cells, those where VEGFR2 siRNA was applied did show a significant decrease in tube length. Values are expressed as mean ± SEM (N ≥ 3). Significance levels: *P* < .05 (*), *P* < .01 (**) and *P* < .001 (***)

VEGFR‐2 became activated (phosphorylated) after adding RPE‐derived exosomes to HUVECs, and its activation increased further when the exosomes added were derived from low‐stressed RPE cells (Figure 5A).

In order to determine whether VEGF was needed for the activation of VEGFR2 upon the addition of exosomes, recombinant soluble Flt1‐Fc, commonly used to trap VEGF, was added to HUVEC cultures together with RPE‐derived exosomes. When RPE‐derived exosomes, from healthy and low‐stressed RPE cells were added to HUVEC cultures, total length of the tubes increased (Figure 5B). Interestingly, when Flt1‐Fc was added to the medium—arresting VEGF—total length of the tubes did not decrease (as it would be expected if it was VEGF‐dependent; Figure 5B), indicating that the positive angiogenic effect of exosomes is VEGF‐independent.

To determine whether the increased tube formation observed is due to the increased activated VEGFR2 observed upon the addition of the exosomes, we inhibited VEGFR2 expression in HUVECs by siRNA (Figure 5C). When tube formation experiments were performed, results showed that ECs treated only with vehicle responded normally to VEGF (Figure 5D, left panel). Contrarily, those HUVEC transfected with the VEGFR2 siRNA did not respond to exosomes (Figure 5D, right panel). Thus, VEGFR2 present in HUVECs is required for mediating the exosomes‐induced tube formation.

### 3.5 Angiogenesis is enhanced by VEGFR2 from RPE‐derived exosomes

**Figure 6:**
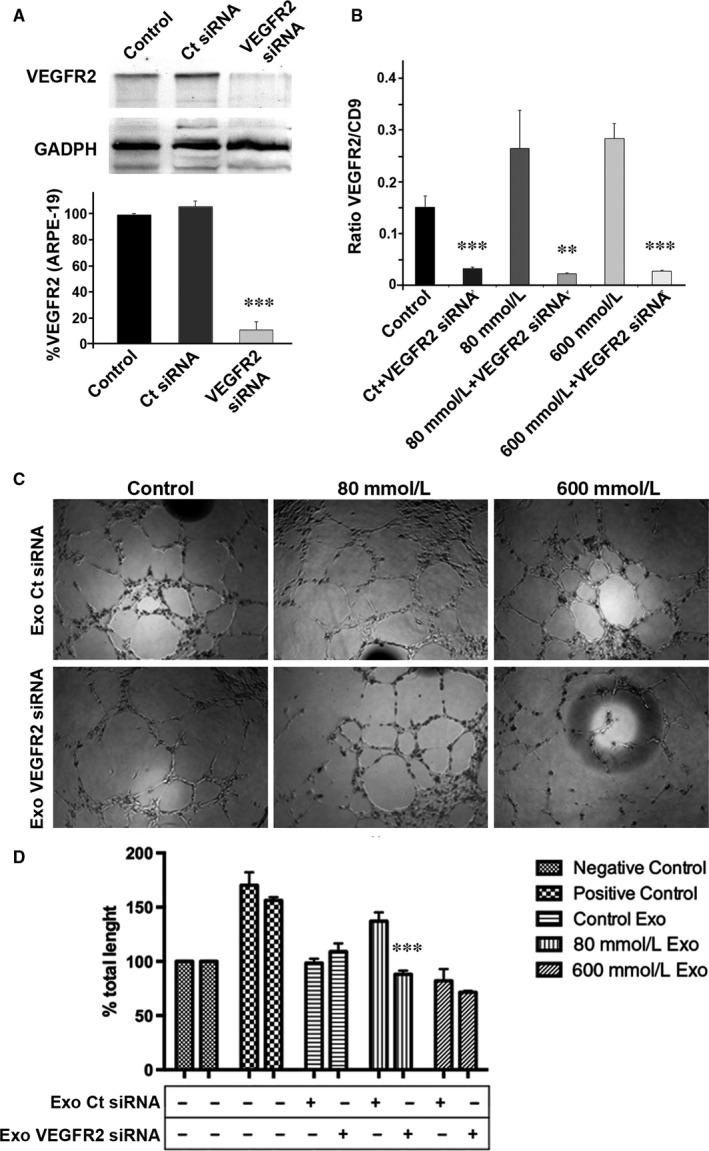
Angiogenesis depends on VEGFR2 from RPE‐released exosomes. A, Blots show expression of VEGFR2 before and after VEGFR2 siRNA was applied to ARPE‐19 cells. The bar chart represents the relative levels of VEGFR2 expression when compared to control. B, When VEGFR2 siRNA was applied in ARPE‐19 cultures, the set of exosomes expressing VEGFR2 was reduced significantly in every situation. C, HUVEC treated with exosomes from RPE cells treated differently form tubes. D, Total length after applying exosomes into HUVEC cultures. When exosomes from low‐stressed RPE cells treated with VEGFR2 siRNA were added to HUVEC cultures, total tube length was significantly reduced. Flow cytometry dot plots available in Figure S4. Values are expressed as mean ± SEM (N ≥ 3). Significance levels: *P* < .01 (**) and *P* < .001 (***)

In order to investigate whether the VEGFR2 derived from the RPE cells is responsible for inducing angiogenesis, we knocked down VEGFR2 in control, low‐stressed and high‐stressed RPE cells (Figure 6A). Subsequently, exosomes were isolated and VEGFR2 levels were quantified (Figures 6B and S6). It was observed that the fraction of exosomes expressing VEGFR2 was dramatically reduced in every case (Figure 6B). EVs were then added to HUVEC, and the formation of new blood vessels was studied afterwards. HUVEC treated with control RPE‐derived exosomes, transfected with siRNA control or with VEGFR2 siRNA, presented no differences in tube formation (Figure 6C, left panels; quantification in Figure 6D). Low‐stressed RPE‐derived exosomes were added to HUVEC cultures. When these exosomes came from RPE cells where VEGFR‐2 had been silenced, new blood vessel growth was significantly lower than those HUVEC nontreated with VEGFR2 siRNA (Figure 6C, centre panels; quantification in Figure 6D). High‐stressed RPE‐derived exosomes applied to HUVEC had the same influence in tube formation, when VEGFR2 siRNA was applied and when it was not (Figure 6C, right panels; quantification in Figure 6D). These observations thus suggest that VEGFR2 from low‐stressed RPE‐derived exosomes is necessary for inducing tube formation.

## 4 DISCUSSION

Overproduction of ROS activates cellular self‐defence mechanisms, but when such production is excessively high, cell death inevitably occurs. It has been repeatedly observed that EtOH exerts an oxidative effect in several tissues, including the RPE.^2^, ^3^, ^37^ Furthermore, moderate levels of EtOH have been observed to enhance angiogenesis by promoting VEGF release.^38^

Enhanced autophagy flux in RPE cells contributes to proper RPE function.^39^ This exceptionally high physiological autophagic activity in RPE cells is mainly due to POS phagocytosis.^40^ Autophagic activity in RPE cells increases after ROS damage induced by EtOH, encouraging formation of more autophagosomes and subsequent autolysosomes. This effect was observed in RPE cells of aged mice and AMD patients.^22^ It was also observed that OS in ARPE‐19 cells does not kill them, but rather enhances autophagy,^2^ VEGF release^41^ and exosome liberation.^19^ In this sense, RPE cells have been reported to express p62 in conditions of autophagy impairment in a collagen XVIII‐deficient mouse model.^42^

Several studies have described the link between autophagy and exosome biogenesis in disease. Bhattacharya described how both mechanisms work together in pancreatic tumour cells.^43^ Wang described how autophagy and RPE exosomes work together in the formation of drusen in AMD.^22^ We have altered autophagy in ARPE‐19 cells by silencing the gene that encodes for Atg7. Atg7 mediates the conjugation of other proteins during autophagy, such as Atg3 and Atg8, which has a number of orthologues in mammals, including LC3.^44^ Thus, after silencing Atg7 with a specific siRNA, the autophagosome cannot be formed. In such an experiment one might expect a higher number of MVBs ready to fuse with the cell membrane and liberate their content, thus releasing exosomes. However, we observed that the number of exosomes was reduced. Thus, it is possible that MVBs were degraded by lysosomes via an independent autophagosome pathway.^23^

It has been previously established that EVs, particularly exosomes, play a central role in the proliferation, migration and tube formation of endothelial cells in different biological systems—in both physiological and pathological scenarios—by either increasing^45^, ^46^ or reducing^47^ angiogenesis. Furthermore, RPE cells seem to release specific exosomes through the basolateral membrane towards the choroidal ECs.^48^, ^49^ These exosomes. An earlier study showed that stressed RPE cells released numerous exosomes expressing VEGFR2 which, when exposed to endothelial cells, hasten their growth.^19^ Since autophagy influences exosome formation, its reduction in stressful conditions might also reduce angiogenesis, as observed in Figure 3. It is well established that VEGFR2 mediates migration of endothelial cells during angiogenesis,^50^ which can be activated by heat shock protein 20 (Hsp20).^51^ Even though physiological development and maintenance of the choriocapillaris requires RPE‐derived VEGF,^52^, ^53^ our observations showed that endothelial cells were still forming tubes when VEGF‐A and ‐B were blocked using a trapper, thus pointing that the pathological mechanism might occur in a VEGF‐independent manner. New blood vessels formed during AMD or DR, like tumour vessels, are abnormal, and it has previously postulated that their growth might occur via alternative VEGF‐A pathways.^54^

Apparently, as aforementioned, stress‐induced abnormal angiogenesis can occur independent of VEGF‐A, and the pathway is not clear yet. What is clear is that VEGFR2 is activated during abnormal angiogenesis and that RPE‐released exosomes are contributing with their own cargo of receptors. When VEGFR2 was inhibited in HUVEC—by means of a VEGFR2 siRNA—tube formation was arrested. When VEGFR2 expression was inhibited in RPE cells and the released exosomes were added to HUVEC cultures in a consistent manner, we observed that tube formation was significantly reduced. These last outcomes point that, even when endogenous endothelial VEGFR2 is critical for the growing of abnormal blood vessels, external input from RPE‐derived exosomes might be decisive for the distinctive angiogenesis observed in neovascular eye diseases, such as AMD and DR, and that this exosome release is directly related to autophagy.

## CONFLICT OF INTEREST

The authors have no conflict of interest.

## AUTHOR CONTRIBUTION

JMB, FJR and JSP designed the research. SAA, GSH, AFV, CRA, MM and JMGV performed the research. SAA, GSH, AFV, CRA and JSP analysed the data. JMB, FJR and JSP wrote the paper.
