# Supplementary figures and images for: Role of retinal pigment epithelium‐derived exosomes and autophagy in new blood vessel formation
Source: J Cell Mol Med. 2018 Aug 21;22(11):5244–56. doi: 10.1111/jcmm.13730 (PMC6201377; doi:10.1111/jcmm.13730)

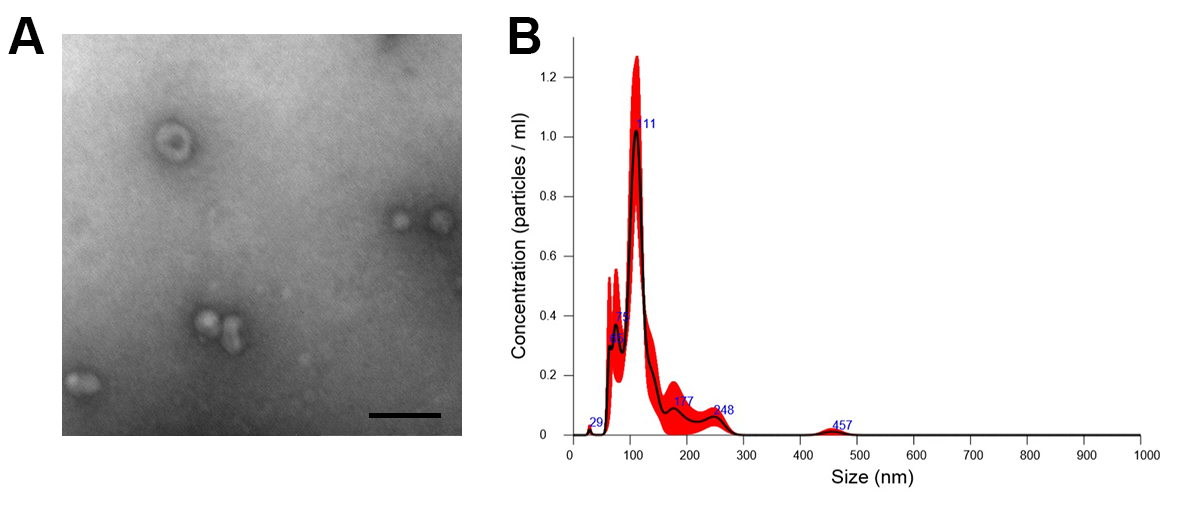

Supplement: Supplementary file 1 [file JCMM-22-5244-s001.tiff]

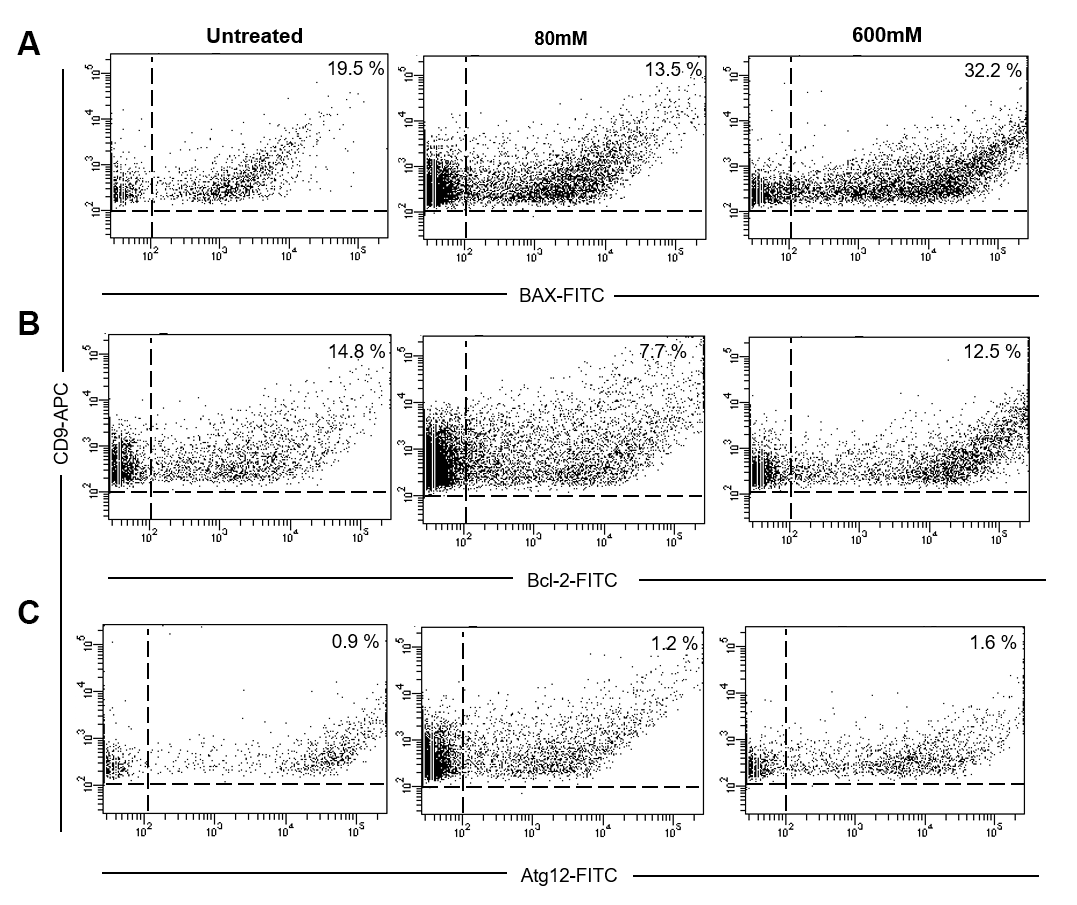

Supplement: Supplementary file 2 [file JCMM-22-5244-s002.tiff]

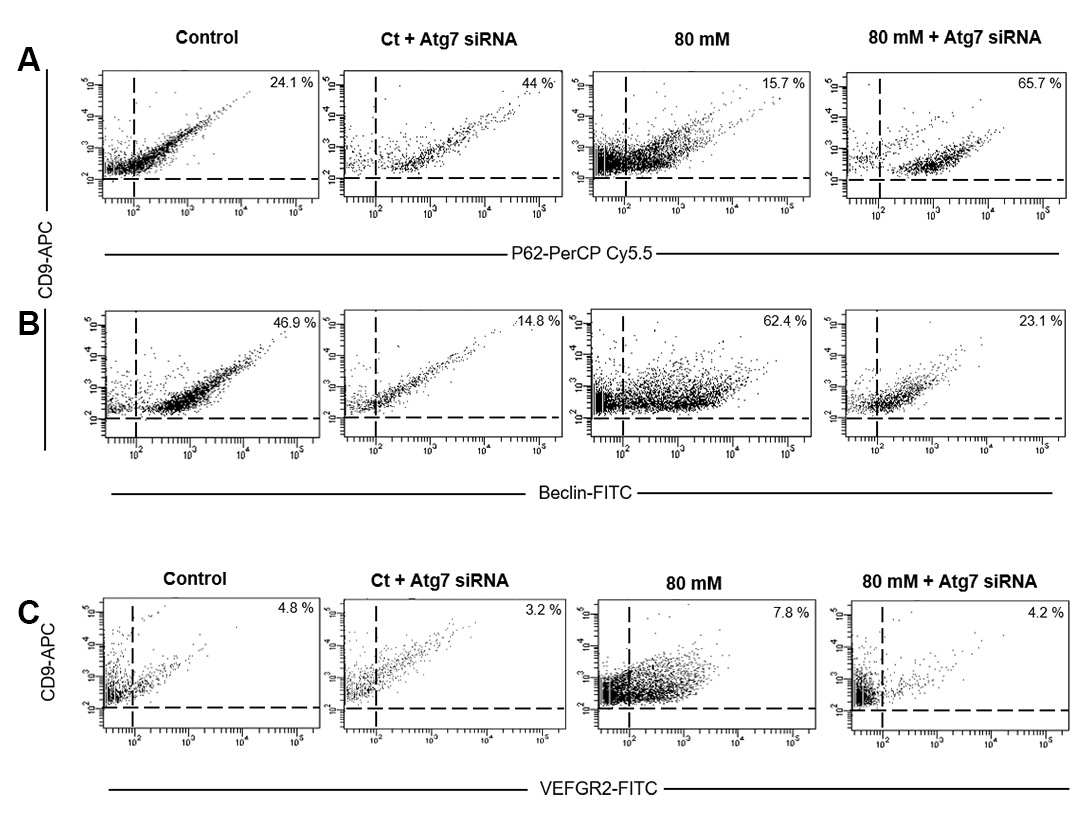

Supplement: Supplementary file 3 [file JCMM-22-5244-s003.tiff]

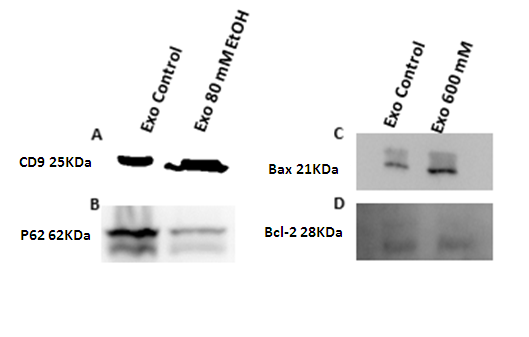

Supplement: Supplementary file 4 [file JCMM-22-5244-s004.tif]

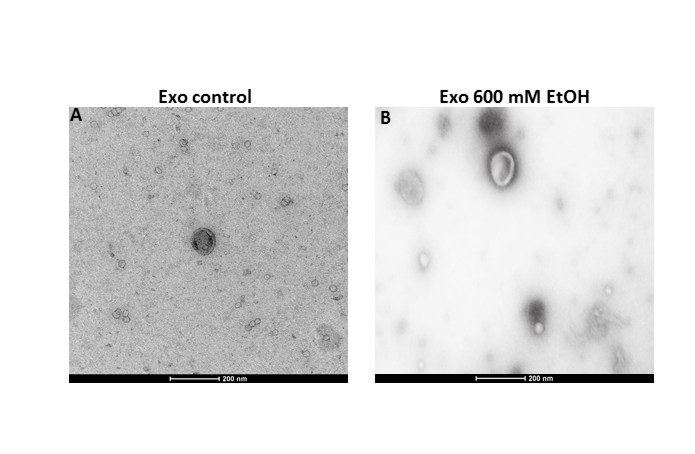

Supplement: Supplementary file 5 [file JCMM-22-5244-s005.tif]

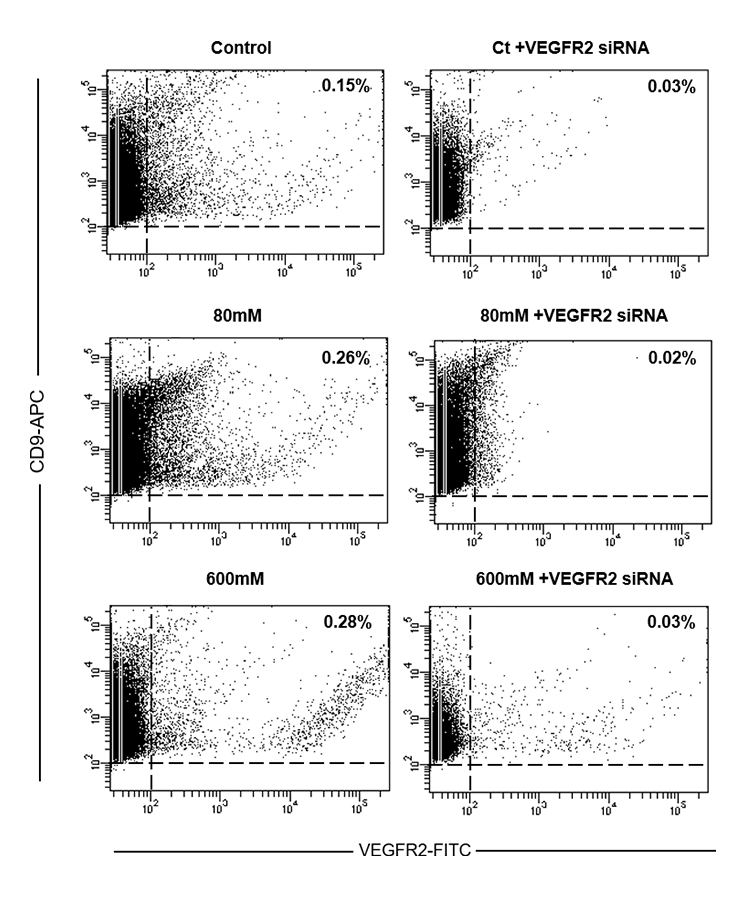

Supplement: Supplementary file 6 [file JCMM-22-5244-s006.tiff]
