# Supplementary material for: Role of retinal pigment epithelium‐derived exosomes and autophagy in new blood vessel formation
Source: J Cell Mol Med. 2018 Aug 21;22(11):5244–56. doi: 10.1111/jcmm.13730 (PMC6201377; doi:10.1111/jcmm.13730)
Supplement: Supplementary file 7 [file JCMM-22-5244-s007.docx]

**Supplementary figure 1. Exosomes released from ARPE-19 cells exhibit the classical morphology and size.** (A) EVs were detected in extracellular medium of ARPE-19 cells treated and untreated. (B) Size-distribution analysis of exosomes was performed by the Nanosight microparticle tracking system, proving that most of the EVs hold typical size of exosomes (50–150 nm). Scale bar in A represent 100 nm.

**Supplementary figure 2. Flow cytometry analyses of exosomes from RPE cells expressing Bax, Bcl-2, and Atg-12.** Flow cytometry analyses of Bax (A), Bcl-2 (B), and Atg-12 (C) expression on CD9-positive exosomes released from untreated and EtOH (80mM and 600mM)-treated ARPE cells. A representative dot plot of at least three experiments showing the percentage of co-labeled exosomes with FITC-antibodies binding to apoptosis or autophagy-related proteins and APC-conjugated CD9.

**Supplementary figure 3. Flow cytometry analyses of exosomes from RPE cells treated with Atg7 siRNA.** The effects of blocking autophagy using small interfering RNA against Atg-7 gene on exosomes secretion in healthy and ARPE-17 cells subjected to low stress induced by EtOH treatment. Representative flow cytometry graphs (n=3 independent experiments) displaying the fractions of exosomes expressing p62, Beclin (A) and VEGFR2 (B) out of the total exosomes collected from culture supernatants of untreated control, Atg7 siRNA treated control, untreated stressed and Atg7 siRNA treated stressed cells. Numbers on the right top indicate percentage of mentioned proteins/CD9 double-positive exosomes.

**Supplementary figure 4. Western blot analysis of CD9, P62, Bax and Bcl-2 in exosomes released from ARPE-19 cells in stress conditions**. Relative expression levels of CD9 and P62 were analyzed by Western Blot in exosomes released from untreated (A) and treated ARPE-19 cells with low (80mM EtOH) stress conditions (B). Relative expression levels of Bax, Bcl-2 in exosomes released from ARPE-19 cells untreated (C) and treated with high (600mM EtOH) stress condition as a positive control (D).

**Supplementary figure 5. Exosomes morphology were confirmed by Electron Microscopy**. Exosomes released from ARPE-19 cells exhibit the classical morphology and size (50–150 nm). They were detected and isolated in fresh extracellular medium from untreated cells (A), and in extracellular medium from treated cells with high concentration of ethanol, as a positive control (B).

**Supplementary figure 6. Flow cytometry analyses of exosomes from RPE cells treated with VEGFR2 siRNA**. Control, 80mM and 600mM EtOH-exposed RPE cells were transfected with siRNA targeting VEGFR2. Following 48h silencing procedure, exosomes secreted in the culture medium by non-silenced and silenced cells were isolated and the presence of the vascular receptor 2 on CD9-positive exosomes were determined by flow cytometry. Percentage (n=3 independent experiments) of VEGFR2/CD9 positive exosomes released by cells under different conditions is shown in the right upper quadrant of each representative dot plot.
